# Determinants of a Variety of Deviant Behaviors: An Analysis of Family Satisfaction, Personality Traits, and Their Relationship to Deviant Behaviors Among Filipino Adolescents

**DOI:** 10.3389/fpsyg.2021.645126

**Published:** 2021-05-05

**Authors:** Angelo Reyes Dullas, Kristine Danielle Yncierto, Mariel A. Labiano, Jerome C. Marcelo

**Affiliations:** Department of Psychology, College of Arts and Social Sciences, Central Luzon State University, Science City of Muñoz, Nueva Ecija, Philippines

**Keywords:** deviant behavior, family satisfaction, personality traits, Filipino adolescents, family structure

## Abstract

In previous decades, numerous involvements of adolescents in deviant behavior have been increasing, and previous researchers examined different variables that may influence these phenomena. This study was designed to look for the possible predictors of deviant behavior, as well as its association with family satisfaction and personality traits. The study was conducted on 1500 participants ages 12–19 years old from selected schools in Nueva Ecija. The researchers used the Deviant Behavior Variety Scale (DBVS) by Sanches et al. (2016). It consists of 19 items (minor and severe) of a variety of deviant action such as thefts, drug and alcohol consumption, verbal and physical aggression, possession of weapons, vandalism, truancy, lies and defiance of authority, and selling drugs among adolescents (Sanches et al., 2016). Out of 1500 samples, 1227 met the criteria for the deviant behavior scale. Descriptive and Inferential statistics such as *Mean*, *sd*, frequency, percentage, Regression analysis, Pearson-correlation, and Mann Whitney U test were used to analyze this study. The research found that there are differences in levels of deviant behavior (Minor and Severe infractions) among sexes. Results showed that female participants have higher tendency to engage in minor infractions of deviant acts, while males had a higher rate of participation in severe infractions of deviant acts. Moreover, there is a negative/inverse association between family satisfaction and deviant behavior. This implies that respondents who participate more in deviant behaviors are found to be less satisfied with their family life, while respondents who participate less in deviant behavior are more satisfied in their family life. Lastly, the current study found that personality trait-agreeableness is found to be the best predictor of deviant behavior among adolescents.

## Introduction

Considering several studies, adolescence has been described as a distressing stage among individuals since it is in the crisis stage, and many difficulties are experienced by teenagers as well as by those people around them (Sanchez-Sandoval and Palacios, 2012). Teenagers experience many changes in their lives such as bodily changes, too many demands of life, adaptation to their surroundings, stress caused by external forces, pressure in school, family, other relatives, and peer pressure. All of this plays a vital role that, if it cannot be prevented, can lead to a serious defiant act among teenagers (Feist et al., 2013).

Moreover, the adolescence stage is an unrestrained time of growth and changes when it comes to their personality. A study has indicated that between being a child, their environment and especially their family are the most significant influences that can shape adolescents’ personalities (Zaky, 2017). While creating their own identity, adolescents may encounter several conflicts, such as learning wrong behavior from significant people and dealing with their problems in the wrong ways. In accordance with Social Psychology, people learn and form their behavior and personality through interaction with their significant people, significant events in their lives, norms, attitudes, and orientations of behavior, regardless of if it is bad or good (Abrams et al., 2010). Since individual first interaction occurs in the family setting, it is important to examine this context. Family relationships and processes and their association with adolescents’ personalities and future outcomes have been the subject in a recent study (Nkhata and Mwale, 2016). Some parents see adolescents’ deviant behavior as an exaggerated way of rebelling and can be manageable in such times. However, these chances are always a risk. If this deviant behavior is not taken seriously, it might lead to some more serious acts such as anti-social behavior or crime.

Idris et al. (2017) defined deviance as any behavior that does not follow expected rules, beliefs, and norms according to the established standard of the society. Hence, there are some acts considered deviant to certain cultures and not to others. Thus, categorizing what is deviant is always based on how society views, takes, or describes deviant action. In addition, Torrente and Vazsonyi (2012) defined deviance as a structure of behavior that is contrary to the accepted norms and appears as unbalanced mental processes, violating self-realization and evading their own moral behavior and identity control. That is, applying unacceptable behavior and not allowing an individual to function effectively with others as a good member of society.

In the Philippine context, frequencies of deviant behavior have grown more serious from a minor to severe form that can be seen in every region, and the alarmingly high crime rates have been widespread throughout the past years. In previous years, high elevated patterns of reported deviant action among adolescents have become more serious. Social Welfare and Development (DSWD) also worried about the alarming rise of Filipino children in conflict with the law. Additionally, published articles of *Philippines crime and safety report* (2017), the most particular among deviant acts of adolescents includes those behaviors that significantly affect other people in bad terms, such as committing crimes and breaking laws; rape, robbery, theft, murder, juvenile delinquency, and assault; in school; bullying, vandalism, addictions, and substance abuse.

Thus, the main goal of the study is to look for the possible predictors of deviant behavior, as well as its association among family satisfaction and personality traits. Additional objectives of the current study are to look for the differences in terms of male and female scores on the Deviant Behavior and Satisfaction with Life Scale (SWLS).

### Family Satisfaction and Deviant Behavior

Based on empirical studies, satisfaction is all about conscious or cognitive evaluation of an individual and assessing their level of life satisfaction and quality of their life using factors such as family relationships, friends, school, based on an acceptable standard of satisfaction of living (Lewis et al., 2011). To expand this, high satisfaction can shield the individual from engaging in risky behavior (Ye et al., 2014). Further, involvements and experiences within the family are two of the most important sources of life satisfaction and negative emotions of the members of the family. Family satisfaction is referred to as the state in which an individual from the structure of the family and the person with whom he or she has relationships (parent–children and siblings), is being satisfied in whatever family structure they have (Berne et al., 2013; Sharaievska and Stodolska, 2016). In recent research on a Philippines setting, it was found out that the main source of happiness and satisfaction among Filipino samples, such as Filipino farmers (Dullas and Acoba, 2013), Filipino farm children (Tolentino and Dullas, 2015), and Filipino Persons with Disabilities (Nebrida and Dullas, 2018) are their family. Moreover, the World Values Survey found out that a satisfied person has strong family relationships (Alesina and Giuliano, 2010). Therefore, individuals with strong family relationships and high family satisfaction are not prone to engaging in risky behavior. This also serves as a protector in negative life events such as emotional distress, risk behavior, violence, poor academic performance, and involvements in alcohol, cigarettes, and drugs (Cavanagh and Huston, 2008). People lacking close family ties are prone to negative vices (Lee et al., 2013). Correspondingly, in collective cultures, pleasant relationships with others are considered more important compared to personal goals (Park et al., 2004). Such that in Filipino culture, a collective culture, there is a higher consideration toward family satisfaction compared to personal goals. Therefore, in certain Filipino adolescents, family support and guidance throughout their lives may significantly influence family satisfaction. Perhaps, an adolescent who is satisfied in their family may be at a lower risk of engaging in deviant behavior. Considering the recent study, it is hypothesized that those individuals who have a lower level of family satisfaction and have had a troubling experience in life might be at risk of cognitive and behavioral problems.

### Personality Traits and Deviant Behavior

Personality traits have been indicated as one of the most significant predictors of individual outcomes and behaviors (Youn and Fumio, 2014; Abdullah and Marican, 2016; Forrester et al., 2016). While ‘traits’ refer to the ways of perceiving, thinking, and behaving toward the environment and oneself (Youn and Fumio, 2014). Personality traits indicate how we cope with stressful experiences in our life (Feist et al., 2013). Stressful life experiences and how people cope with them play a vital and important role for certain behavior (Aleksic and Vukovi, 2018). On the other hand, Castillo (2017) stated that personality traits could be defined as the way an individual’s mental world is set in a world that is stable and consistent over time. When it comes to personality traits, one of the most famous and useful models is the Big five model (McCrae and John, 1992) in diverse cultures like American-English, Czech, Dutch, Flemish, Filipino, German, Hungarian, Italian, Japanese, Romanian, and Russian (de Raad, 2000). The following are the five personality traits that will be defined.

*Openness to experience* is defined as the openness of an individual which is viewed as willingness to look for new experiences and embrace changes, level creativity, and exploration of unfamiliar things (McCrae and John, 1992; Castillo, 2017). Furthermore, individuals with high potential to be open to experiences are considered as willing to experience changes and new adventures in their lives (Jia et al., 2013).

*Conscientiousness* is defined as traits that include: dependability, capability, accomplishment striving, accountability, self-control, and efficacy (McCrae and John, 1992; Mathisen et al., 2011). Meanwhile, individuals with a high level of conscientiousness are viewed as well organized, good planners, achievement-oriented, have greater job satisfaction, and have positive social relationships (Vardi and Weitz, 2004). Individuals with high conscientiousness are inclined to be hard-working, well-organized, reliable, trustworthy, and firm; and those with low conscientiousness tend to be lazy, disorganized, unreliable, untrustworthy, and indecisive. On the contrary, Krueger et al. (1996), presented that those unconscientious people exhibit criminal tendencies and link to positive attitudes toward delinquency.

*Extraversion* measures the interpersonal interaction of individuals toward other people in the form of being outgoing or shy, as well as the capacity for joy (Castillo, 2017). Additionally, those individuals high in extraversion are sociable, outgoing, assertive, un-reserved, and companionable; those low in extraversion tend to be quiet, reserved, and timid (McCrae and Costa, 1992; Forrester et al., 2016). Low levels of extraversion, which is introversion, showed uncomfortableness, shyness, unsociability, and reserved personality (Feist et al., 2013). There have also been studies that showed evidence that extraversion is significantly linked to maladaptive, antisocial, and deviant behavior (Prinzie et al., 2010; Abdullah and Marican, 2016).

*Agreeableness* refers to the level of compassion within the self to the resentment of an individual, and to the different effects and behavior involved. Bowling and Eschleman (2010) stated that agreeable persons are viewed to be pleasant, tolerant, helpful to others, trusting, forgiving, considerate to others, and cooperative. A tendency toward high agreeableness is much more likely to be considered as more cooperative, understanding, warm, sincere, well-mannered, naturally good, compassionate, friendly, and sympathetic; a tendency toward low agreeableness is more likely to be harsh, rude, cold, unsociable, insincere, and unsympathetic (Forrester et al., 2016). Further, some studies have called low agreeable people unconcerned with others, and therefore can be unfriendly and uncooperative, independent and have poor personal affections toward others (Bodankin and Tziner, 2009). This type of personality (low agreeable) might lead individuals to engage in deviant behavior and crimes.

*Neuroticism* assesses an individual’s emotional instability, such a psychological distress, unrealistic ideas, and maladaptive coping response and can lead to an individual being tense, insecure, irritable, and having grandiose characteristics; people with low neuroticism tend to be calm, self-confident, and patient (McCrae and Costa, 1992; Forrester et al., 2016).

The associations of the five factors model still have a lot areas that need to be explored. There are only a few studies that exhibit adolescents’ personality traits and their association in engaging deviant behavior. Thus, this paper aimed to look further into this domain and its relationship to deviant behavior.

### Theoretical Framework: Five Factor Theory

McCrae and Costa’s (1992) five factor theory (FFT) discusses these concepts. Basic Tendencies are materials of traits and essences that serve as measurements of the consistency of behavior across situations. Since personality traits are biologically based, they are universal and developed throughout time. Thus, researchers would like to know if personality traits have something to do with the engagement of adolescents into deviance.

Characteristics Adaptations refers to acquired characteristics as affected by conditions and their capacity to be influenced by external influences (Novikova, 2013). What a certain person has learned, an individuals’ acquired and specific skills, are characteristic adaptations. Characteristic adaptation differs from culture to culture, and allows an individual to fit into their environment on an ongoing basis (McCrae and Costa, 1992). Therefore, the deviant tendencies of adolescents are considered as characteristic adaptations since they possess the accountability to become one.

Self-concept refers to knowledge and attitude about oneself, also part of characteristic adaptation. It is an important adaptation that consists of how an individual gains a sense of purpose and views their life (Feist et al., 2013). Self-concept is one’s belief in his or her own purpose and awareness of oneself. Thus, in this study, the level of family satisfaction is considered as self-concept.

### Current Study

Based on past literature, several factors are linked to deviant behaviors of adolescents such as family factors and peer pressures (Khodarahimi, 2013; Idris et al., 2017). In the Philippine setting, Deviant behavior among youth is increasing which includes incidents like drugs, smoking, alcohol, vandalism, school dishonesty, and crimes such as rape, murder, theft, and juvenile delinquency.

Thus, the major research objective is to find the determinant of deviant behavior among Filipino adolescents using variables such as personality traits, family structures, and level of family satisfaction. Additional objectives of the current study are to look for the differences in terms of male and female scores on the Deviant Behavior and Satisfaction with Life Scale (SWLS). However, a major delimitation of the study is the usage of self-report.

## Methodology

### Research Design

**FIGURE 1:**
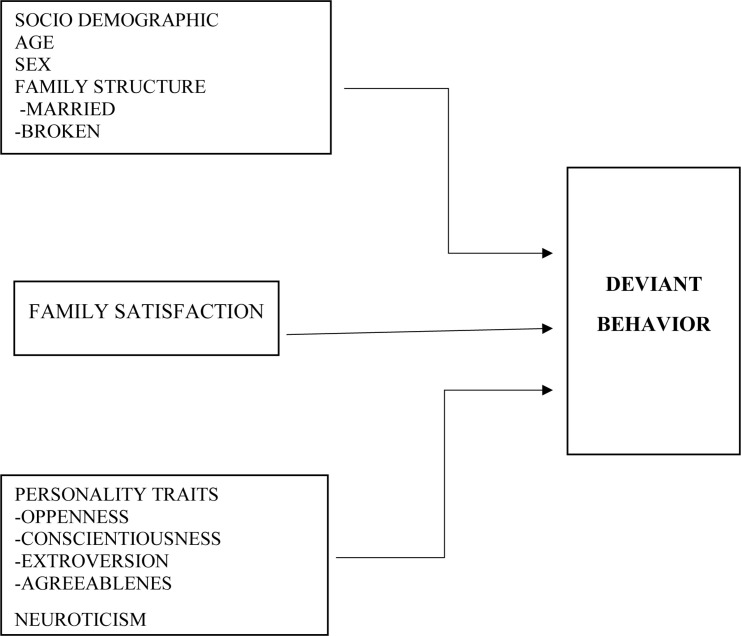
Conceptual paradigm.

The research utilized correlation using Multiple Regression Analysis in exploring predictors of deviant behavior using the level of family satisfaction, personality traits, and socio demographic information (age and family structure). The conceptual representation of the variables of the study are provided in Figure 1.

### Participants

Filipino adolescents are the sample of the study, ages 12–19 years old. They were selected from first-year high school (grade 7) to first-year college in Nueva Ecija. The sample consisted of 1500 participants. Purposive sampling was used for the selection of the participants. Age was used as continuous data.

### Sampling Procedure

The current study used cluster sampling among the list of schools (high school and colleges) in Nueva Ecija from 19 high schools and 20 colleges and universities. The schools were randomly selected. After clustering the high schools and colleges, selection from the list of schools was randomized for the equal opportunity of being selected. From 39 high schools and colleges, a sample consisted of 1500 participants. Researchers purposively selected the respondents within an age range of 12–19 years old and it was required to be a student in each school in Nueva Ecija. Out of 1500 participants, only 1227 participants passed the criterion for deviant behavior.

### Instrumentation

The questionnaire consists of three sections that include basic data such as the deviant behavior scale, family satisfaction scale, and personality traits inventory (see Appendix). The Deviant Behavior Variety Scale or DBVS (Sanches et al., 2016) consists of 19 items of a variety of deviant actions such as thefts, drug and alcohol drinking, and other risky behaviors among adolescents. The questionnaire contained two levels of deviant behavior, the minor infractions (MI) and severe infractions in accordance with the seriousness of the act. Each item was expected to represent different kinds of infractions. The DBVS has a high internal consistency (Cronbach α = 0.829). Moreover, in an undergraduate thesis study of Villanueva et al. (2018), the reliability of the scale with the use of Cronbach α, showed that the scale scored α = 0.96 indicating a high level of internal consistency. Therefore, DBVS showed a high consistency across different situations or settings across the region.

The Satisfaction with Family Life scale or SWFL (Zabriskie and Ward, 2013), is made of five sentences in the form of a 7-point Likert scale about satisfaction with life. Zabriskie and Ward (2013) reported evidence of high internal consistency. Also, scale usability is high since it used basic language and simple instruction about family satisfaction. Therefore, the author (Zabriskie and Ward, 2013) reported a high consistency of the SWFL scale among all samples suggesting that it can be used worldwide in measuring family satisfaction across time, places, and different cultures.

The Big Five Inventory or BFI is a 5-point scale which measures Agreeableness, Extraversion, Conscientiousness, Neuroticism, and Openness domains. It has high reliability measures (α = 0.75–0.80) and high-test retest reliability (α = 0.80–0.90) (John and Srivastava, 1999).

### Data Gathering Procedure

The researchers go to the selected schools (high schools and colleges) from a randomization of schools in Nueva Ecija. A letter of introduction to request permission before conducting the main study in schools was first disseminated to the faculty and school administration. After letting the researchers conduct for their purpose, the researchers then purposively selected respondents with an age range of 12–19 years old. The researchers first introduced to the respondents their purpose before conducting the data. Respondents then completed all three questionnaires in one session. The respondents informed the participants that data was analyzed individually, and respondents’ feedback on the results is possible.

After conducting all data, the researchers filtered out the data. The criterion for this was that scores ranging from 1 to 19 were indicated as engaging in deviant acts, and sores of zero (0) from the group were out. Researchers considered the participants’ (1227) socio-demographic data (age, sex, and family structures), level of family satisfaction, as well as their personality traits.

### Ethical Consideration

All ethical aspects were acquired in relation to participants’ safety. This paper was conducted with the voluntary participation of every respondent, and researchers informed all participants that they have the rights to withdraw their data anytime if they did not feel comfortable allowing it to be used in object of the current study. Participants were also informed about the questionnaire, data protection was also applied, the data collected stayed anonymous, and strictly confidential data was obtained. Researchers used informed consent (see Appendix) for the administration of the school and for the student itself. There was no use of deception nor manipulation of the data collected from respondents. All the data obtained remained true to its original form. Lastly, the questionnaires that were used in this study were standardized, tested, and validated among famous researchers.

### Data Analysis

Descriptive statistics such as *Mean*, *sd*, and frequency were used to analyze the sociodemographic characteristics of the participants. Inferential statistics such as Pearson-moment correlation and regression analysis were used to find out determinants of deviant behavior among Filipino adolescents using variables such as personality traits, family structures, and level of family satisfaction. In determining the difference between sexes and family structures, a *Mann–Whitney U* test was used in this study. Correlational analysis was also used to determine the relationship between family satisfaction and deviant behavior; personality traits and deviant behavior; and age and deviant behavior. For analysis of the regression models, independent variables such as age, family satisfaction, and personality traits (agreeableness, neuroticism, openness to experience) serve as factors to deviant behavior for the identification of the best predictor/s. If one of the variables did not possess a significant contribution for predicting the dependent variable (deviant behavior), then it was removed depending on the criterion of the *p*-value. The cutoff value will be at a level of significance greater than 0.05.

## Results

In this part, the researchers studied the relationship of deviant behavior, family structure, family satisfaction, and personality traits. The interactions of every variable within each other are tabulated below. The table includes the two levels of deviant behavior, the differences between male and female, the relationship of family structures and deviant behavior, the relationship of personality traits and deviant behavior. The last table indicates the best predictor of deviant behavior.

The results in Table 1 and Appendices present the socio-demographics of the participants. Results showed that the mean age of participants is 16.74. The highest frequency in terms of age bracket is 18 years old or 26.7% of the total sampling size (*n* = 327) followed by 19 years old or 18.3% (*n* = 225), 17 years old or 15.2% (*n* = 187), 16 years old or 13.7% (*n* = 168), 15 years old or 10.2% (*n* = 125), 14 years old or 9.9% (*n* = 121), 13 years old or 4.5% (*n* = 55), and 12 years old or 1.5% (*n* = 19). In terms of sex, female (628) has a higher frequency than male (599). With regards to family structure, participants with Married family has 809 (65.9%) while participants with separated parents are 417 (34.0%).

**TABLE 1:** Descriptive statistics of the sociodemographic characteristics of the participants.

	**Mean**	**Frequency**	**Percent**
Age	16.74		
12		19	1.5
13		55	4.5
14		121	9.9
15		125	10.2
16		168	13.7
17		187	15.2
18		327	26.7
19		225	18.3
**Sex**			
Male		599	51.2
Female		628	48.8
**Family type**			
Broken		809	65.9
Married		417	34.0
Total	1227	1227	

Table 2 represents the *M* and *sd* of the total deviant behavior among adolescents and the two level-infractions of deviant behavior, which are MI and severe infractions (SI) between males and females. Total deviant behavior in all participants is shown (*M* = 0.218; *SD* = 0.138). It also represents that the mean of minor infraction among females (*M* = 0.366; *SD* = 0.184) was scored higher than the mean of males (*M* = 0.325; *SD* = 0.184). On the other hand, in severe infractions, it resulted higher mean of male (*M* = 0.151; *SD* = 0.110) compared to female (*M* = 0.098; *SD* = 0.110). The result indicated that females are more engaged in MI of a variety deviant acts compared to males, while the male respondents are more engaged in severe infractions of a variety of deviant acts compared to females.

**TABLE 2:** Level of deviant behavior, minor infraction (MI) and Severe Infraction (SI) among male and female.

	***N***	**Mean**	***SD***
**Sex**			
Male	599		
*Minor infractions*		0.325	0.184
*Severe infractions*		0.151	0.110
Female	628		
*Minor infractions*		0.366	0.205
*Severe infractions*		0.098	0.144
Total deviant	1227	0.218	0.138

Table 3 and Appendices shows the comparison of minor and severe infractions in male and female adolescents on the variety of deviant behavior. The researchers used a Mann–Whitney *U* test to determine if the difference is significant. Results showed that male has a higher score (*Mean Rank* = 685.98) than their female counterpart (*Mean Rank* = 545.34). In this sense, females have a higher tendency to engage in MI while the male has a higher tendency to engage in severe infractions (*U* = 144968.500, *p* = 0.000). This indicates males have higher deviant behavior tendencies than females.

**TABLE 3:** Mean of the deviant behavior and the family satisfaction in terms of sex.

**DB**	**Mean**	***SD***	**Description**	**SFLS**	**Mean**	***SD***	**Description**
**Sex**							
Male	0.247	0.147	High		4.84	1.24	Low
Female	0.190	0.121	Low		5.12	1.24	High
**Deviant behavior**	***N***	**Mean rank**					
Male	599	685.98					
Female	628	545.34					
Mann–Whitney *U*	**144968.500**						
Sig (0.01)	**0.000**						
**SFLS**							
Male	599	580.53					
Female	628	645.34					
Mann–Whitney *U*	**168036.500**						
Sig (0.01)	**0.001**						

*The bold values are the significant results of the study.*

Table 3 also presents the comparison between males and females in terms of their satisfaction with family. Results showed that females had higher scores (*Mean Rank* = 645.34) than males (*Mean Rank* = 580.53) with a very high level of significance at 0.000 (*U* = 168036.500, *p* = 0.001). This indicates that females had a higher level of perceived family satisfaction than their male counterpart.

The Table 4 and Appendices represents family structure differences in terms of Deviant Behavior and Family Satisfaction. Results showed that participants who reside within married households have deviant behavior of *M* = 0.183, SD = 0.113, while participants who reside in broken families showed deviant behavior of *M* = 0.283, *SD* = 0.155. For family satisfaction, on the other hand, results showed that participants who reside within married households have a score of (*M* = 0.5.25, *SD* = 1.22), while participants who reside in broken families scored (*M* = 4.48, *SD* = 1.41). To validate the descriptive score, a Mann–Whitney *U* test was used. The descriptive statistics was validated by the results of the Mann–Whitney *U* test.

**TABLE 4:** Mean of the deviant behavior and the family satisfaction in terms of family structure.

**DB**	**Mean**	***SD***	**Description**	**SFLS Mean**	***SD***	**Description**
**Family structure**						
Married	0.183	0.113	Low	5.25	1.22	High
Broken	0.283	0.155	High	4.48	1.41	Low
**Deviant behavior**	***N***	**Mean rank**				
Married	809	532.54				
Broken	417	770.56				
Mann–Whitney *U*	103181.000					
Sig (0.01)	**0.000**					
**SFLS**	***N***	**Mean Rank**				
Married	809	681.76				
Broken	417	481.08				
Mann–Whitney U	113457.000					
Sig (0.01)	**0.000**					

*The bold values are the significant results of the study.*

The results showed that participants with married parents has lower scores (*Mean Rank* = 532.54) on Deviant Behavior measure than participants with broken families (*Mean Rank* = 770.56). The result has very high significance at.000 alpha level (*U* = 144968.500, *p* = 0.000). This implies that participants who reside with married parents have a low tendency to participate in deviant behavior. On the other hand, participants with married parents are also more satisfied with their family (*Mean Rank* = 681.76) than those with broken families (*Mean Rank* = 481.08). The result is highly significant at (*U* = 113457.000, *p* = 0.000).

The result of the main relationship between family satisfaction and deviant behavior showed inverse moderate correlation *(r* = –0.402*; p* = 0.000). This implies that participants who have low levels of family satisfaction tend to be engaged more in deviant acts and *vice versa*.

**FIGURE 2:**
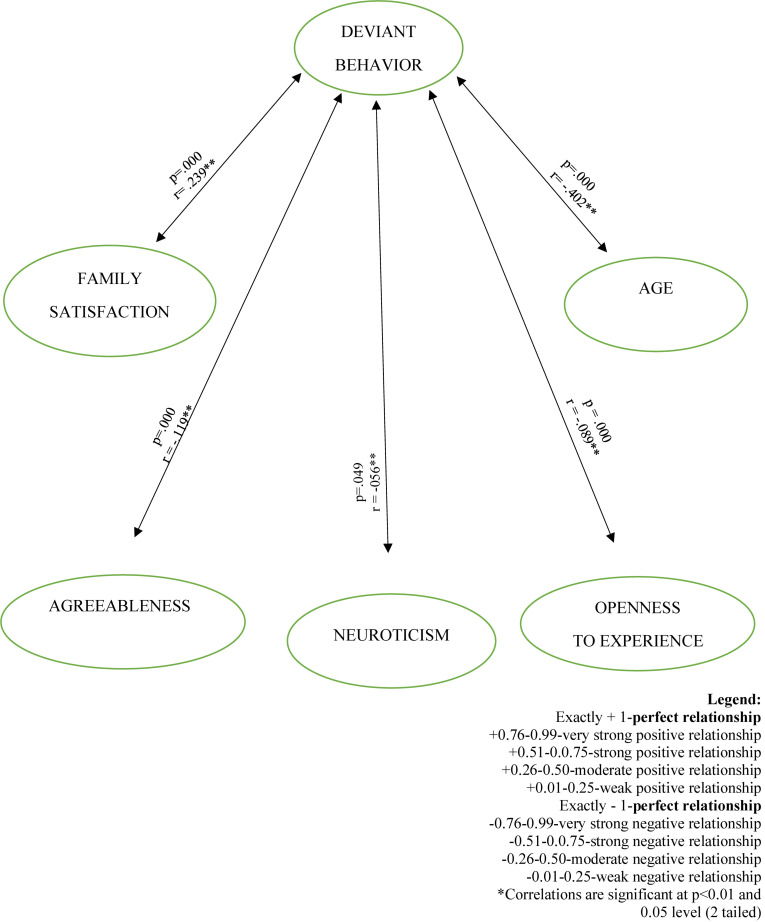
Path analysis of deviant behavior, age, family satisfaction, and personality traits. Exactly + 1-**perfect relationship.** +0.76-0.99-very strong positive relationship. +0.51-0.0.75-strong positive relationship. +0.26-0.50-moderate positive relationship. +0.01–0.25-weak positive relationship. Exactly - 1-**perfect relationship. –**0.76–0.99-very strong negative relationship. **–**0.51–0.0.75-strong negative relationship. **–**0.26–0.50-moderate negative relationship. **–**0.01–0.25-weak negative relationship. *Correlations are significant at *p* < 0.01 and 0.05 level (2 tailed).

Table 5 and Appendices shows a statistically significant correlation between the variables of the study. This indicates that deviant behavior has a linear relationship with age (*r* = 0.239; *p* = 0.000). Also, personality traits of agreeableness possessed a highly significant negative correlation with deviant behavior (*r* = –0.119; *p* = 0.000), and a significant relationship with openness (*r* = –0.089; *p* = 0.002). Thus, as the age of participants increases, there is also tendency for an increased level of their engagement in deviant behavior. In personality traits, agreeableness stands out most of all. However, it indicates that participants with a low level of agreeableness are more likely to engage in deviant acts, while participants who rate high on agreeableness are not prone. The presentation of correlations of the different variables of the study using Path Analysis is provided in Figure 2.

**TABLE 5:** Relationship of deviant behavior to personality traits, family satisfaction and age.

**1**	**2**	**3**	**4**	**5**	**6**	**7**	**8**
(l) MeanDEVIANCE 1	–	–	–0.014	–	–	–	0.239**
	0.832	**0.000**	0.635	**0.049**	**0.002**	**0.000**	**0.000**
(2) MeanEXTROVERSION	1	0.313**	0.419**	0.152**	0.396**	0.144**	–0.096**
		0.000	0.000	0.000	0.000	0.000	0.001
(3) MeanAGREEABLENESS		**1**	0.484^**^	0.100^**^	0.383^**^	0.159^**^	**0.014**
			**0.000**	**0.000**	**0.000**	**0.000**	**0.000**
(4) MeanCONCENTIOUSNESS			1	0.196**	0.441**	0.135**	–0.055
				0.000	0.000	0.000	0.052
(5) MeanNEUROTICISM				1	0.253** 0.000	0.162** 0.000	–0.078** 0.000
(6) MeanOPENNESS					1	0.257** 0.000	–0.057* 0.000
(7)Mean of satisfaction						1	–0.088*
with life scale							**0.002**
(8) Age							**1**

*p < 0.05, **p < 0.01. The bold values are the significant results of the study.

The regression analysis using five factor traits and family satisfaction as predictors explained 16.6% of the variance in deviant behavior shown in Tables 6, 7 and Appendices. To determine whether the personality traits and family satisfaction predict deviant behavior, five factors traits (with the exception of extroversion and conscientiousness since they do not possess the requirements of correlation in deviant behavior), as well as family satisfaction were regressed in Tables 6, 7. The results showed that as deviant behavior increases, 2.0% of personality traits of agreeableness decrease (β= –0.020*; p* = 0.012). Personality traits of openness to experience have no significant influence to deviant behavior (β= 0.011*; p* = 0.173) as well as neuroticism (β= 0.002*; p* = 0.831). Finally, as family satisfaction increases by 4.1%, deviant behavior decreases (β= –0.04*1; p* = 0.000). The Model (R^2^ = 16.6; *F* = 217.803; *p* = 0.000) presents the best predictor of Deviant Behavior.

**TABLE 6:** Model summary of regression of deviant behavior, personality traits and family satisfaction.

***R***	***R* square**	**Adjusted *R* square**	**Standard error of the estimate**	***R* square change**	**df1**	**df2**	***p*-value**
0.408	0.166	0.163	0.12603	0.149	1	1224	0.000

*d. Predictors: (Constant), agreeableness, openness to experience, neuroticism, family satisfaction.*

**TABLE 7:** Relationship of deviant behavior, personality traits, and family satisfaction.

**Variables**	***β***	***T***	***P***	***R***	***R*^2^**	***F***
				0.408	0.166	217.803
Agreeableness	–0.020	–2.504	0.012			
Openness to	0.011	1.365	0.173			
experience						
Neuroticism	0.002	0.213	0.831			
Family satisfaction	–0.041	–14.758	0.000			

*Dependent variable: deviant behavior.*

## Discussion

### Level of Deviant Behavior Among Filipino Adolescents

The results indicated that a variety of deviant behavior is observed among selected Filipino adolescents. The results indicate that the participants are engaging in *MI* which means that they are doing minor deviant acts. These *MI* deviant acts may include drinking alcohol, lying to adults, using public transport without paying, skipping school for several days without the parents’ knowledge, and painting graffiti on buildings or other locations (e.g., school, public transports, walls, etc.). The findings were supported by the past studies of Hanımoglu (2018), which stated that there are high numbers of participation of adolescents in deviant behavior compared to other ages. Some factors that seemingly influence adolescents’ deviant behavior are the changes in their psychosocial, physical body, cognitive and behavioral attitudes that make them more prone to norm-breaking behavior. According to Damron-Bell (2011), identifying vices is motivated by the peer pressure, curiosity, and the idea of being mature. Participation of youth in deviant behaviors maybe characterized by impulsiveness and recklessness of actions. To further explore, this study hypothesized that adolescents’ participation on deviant acts are highly influence by several variables including family structure, family satisfaction, and personality traits, which will be discussed in the next section.

### Deviant Behavior and Family Satisfaction Level of Mean and Standard Deviation Between Sex and Family Structure

Based on the results, male participants have a low level of family satisfaction and have a higher tendency to participate in a variety of deviant acts. On the other hand, female participants attained a higher level of family satisfaction and low tendency to engage in deviant behavior. These results were consistent with the past studies indicating that male has higher tendency in risk taking behavior or simply deviant acts than their female counterpart (Piquero and Hickman, 2001). In a meta-analysis study exploring the differences of gender in deviance, a differentiation was made between females who thought the act was thrilling and risky were prone to engage deviant acts, and female who held morality against deviant acts and were less likely to engage. On the other hand, males who had peers who participated in deviant action were prone to deviant conducts (Piquero and Hickman, 2001). Therefore, participation of deviant acts depends on certain values held and learned through their environment by both males and females.

In the context of comparison between participants with married parents and participants with broken families and their engagement in deviant acts and level of family satisfaction, results indicated that participants’ with broken families engaged more often in severe and MI of deviant acts and have low levels of family satisfaction compared to participants with married parents. It is reported that there is a high variance of deviance among adolescents raised from broken families (Apel and Kaukinen, 2017). With these, Apel and Kaukinen (2017) indicated that individuals who came from broken families may experience weakened bonds from parents as well siblings and have less time to spend on high-quality family activities and be at a high risk of engaging in deviant acts. Further, Amato (2012) specified that adolescents with broken families have an increasing tendency to experience difficulty with social communication, causing these individuals to turn to deviant acts. In addition, Hoskins (2014) found out that a two parenting style shows more parental monitoring compared to other family types. Thus, the more time spent by parents with their offspring leads to less participation in crimes and deviant acts (Keijsers et al., 2010).

### Relationship of Family Satisfaction and Deviant Behavior

This part of the study discusses the relationship between family satisfaction and deviant behaviors of the participants. Results showed a highly significant and an inversed moderate relationship within family satisfaction and deviant behavior. It implies that participants who rated high on family satisfaction are less likely to engage in deviant acts. While on the other side, participants who are less satisfied with their family life are prone to deviant conduct. This result is also consistent with an earlier study about parental and deviant tendencies (Ochoa et al., 2007), that those who have a low level of family satisfaction have a high level of tendency to engage in deviant acts which can also be attributed to the absence of communication patterns (Tanusree and Mukherjee, 2007) and usage of vices such as alcohol (Birhanu et al., 2014). On the other hand, participants with a high level of family satisfaction are less likely to engage in deviant acts. This suggests that individuals who reside in married-parent households received more attention and support (Ochoa et al., 2007) and shows more parental monitoring (Hoskins, 2014).

### Relationship of Personality Traits and Deviant Behavior and Its Implication to Five Factor Theory

The results of this study found no statistically significant relationships between the personality traits extroversion and conscientiousness and surprisingly, low statistical significance and with weak relationship to neuroticism. However, a moderate significant inverse relationship with openness to experience and high significant inverse relationship in agreeableness.

Personality emphasizes that personality bases are within individuals and that personality is quite consistent in diverse situations (Bolton et al., 2010). Lim et al. (2016) indicated that personality traits are useful for predicting future behavior. Additionally, past literature proposed that personality influences social behavior and socialization, as well as social ethics. Below are the possible explanations to these findings.

#### Openness to Experience

Based on the results, a highly significant and inverse relationship of openness to experience and deviant behavior were found. This indicated that participants who rated high on personality traits of openness to experience or the willingness to experience changes and new adventures in life are not prone to deviant conduct (Jia et al., 2013). In addition, the results implied that adolescents who rate high on this personality trait possessed a wide interest, were imaginative, intelligent, curious, inventive, clever, resourceful, and civilized (John and Srivastava, 1999).

#### Conscientiousness

Based on the results, conscientiousness has a not-significant and weak relationship to deviant behavior. The result contradicts the prevailing studies on deviant behavior and conscientiousness. The findings of these studies (Mathisen et al., 2011; Farhadi et al., 2012; Jia et al., 2013; Aleksic and Vukovi, 2018) discusses that conscientiousness has a significant negative relationship with deviant behavior which suggests that people with low conscientious personality traits participate more in variety of deviant acts.

#### Extroversion

Results found that extroversion is not significantly correlated with deviant behavior. This is consistent with the studies of Farhadi et al. (2012) and Aleksic and Vukovi (2018) who stated the non-significance of the relationship between extroversion and deviant behavior.

On the contrary, Santos and Eger (2014), Abdullah and Marican (2016) and Lim et al. (2016), revealed extraversion as a valid predictor of deviant behavior, which associates it with the probability to participate in deviant acts. This is another gray area in the study of extraversion and deviant behavior since conflicting results were found.

#### Neuroticism

Neuroticism and its correlation to deviant behavior resulted in a low and weak significant relationship. This was supported by the study of Farhadi et al. (2012) and Aleksic and Vukovi (2018) who found out that neuroticism has no connection with deviant behavior. Contrary to the studies of Jia et al. (2013) and Abdullah and Marican (2016) which indicates that people high on this personality trait are inclined to experience negative emotions which can be correlated to conflict and may lead to deviant acts, the result of the research stated otherwise. Another contradicting result that can be explored by future researchers.

##### Personality trait of agreeableness as a best predictor of deviant behavior

The model of regression reveals that agreeableness explains greater elucidation in predicting deviant behavior. There is a high and inverse correlation between agreeableness and deviant behavior. In a sense a weak relationship implied that agreeableness is not perfectly predictive of deviant behavior among adolescents, but since it is significant it might have some influence. This means that a highly agreeable person has the tendency to get along with other people, while a low agreeable person might find it difficult to interact well with other people. According to John and Srivastava (1999), a highly agreeable person has characteristics of being sympathetic, kind, pleasing, friendly, cooperative, helpful, and trusting. While on the other hand, people with low agreeability are found to be fault-finding, cold, unfriendly, selfish, quarrelsome, and cruel. The current study was supported by previous studies (Bolton et al., 2010; Farhadi et al., 2012; Nurul et al., 2013; Aleksic and Vukovi, 2018) which clarify that there is an inverse correlation between agreeableness and deviant behavior. Hence, an individual who possesses a low level of agreeableness is more likely to exhibit aggressive behavior and more eager to enter a conflict, thus more likely participate in deviant acts. Additionally, Bolton et al. (2010) and Nurul et al. (2013), also supporting the current study, specified that highly agreeable people are possible to demonstrate lower deviant acts. It might not be surprising given the fact that people who score high in agreeableness are most likely to withdraw from social conflicts and try to avoid situations that they do not find this harmonious, while those low in agreeability demonstrate higher deviant behaviors. Further, Mathisen et al. (2011) indicated that low agreeableness is characterized by the traits of being rude, cold, uncaring, unsympathetic, and bullying. They may also display more aggressiveness and be more eager to have conflict with his or her environments, thus more likely to participate in a variety of deviant acts.

## Conclusion

The major contribution of the research is the usage of big five model of personality and family satisfaction as a predictor of deviant behavior. In addition, this study explored the family structure (married and broken family) among respondents who are prone to deviant behavior. Based on the findings of the results, personality traits of agreeableness are most likely to predict deviant behavior among adolescents. Additionally, low family satisfaction is also a contributor in deviant behavior among adolescents. Moreover, male participants have a higher tendency to engage in deviant acts while female participants are more satisfied with their family than their male counterparts.

### Recommendation

Current research suggests continuing to study the variable personality traits and their relationships to a variety of deviant behavior, given its inconsistent results with past research. In addition, other strategies are needed to examine the relationship between other personality traits such as extraversion, neuroticism, and conscientiousness with deviant behavior. It is suggested to consider both situational and personal factors, such as relationships with other members in family (such sibling relationships). The researchers also suggest using the findings on policy making and family intervention since it involves deviant behaviors of adolescents and its predictors.

Future researchers should include cross sectional studies which compare age brackets such as middle age and old age, as well as consider analyzing interactions among them and different situational factors. In addition, researchers highly suggest studying intercultural factors to explore the variability of predictors of deviant behaviors. Moreover, since the study used self-reporting and was retrospective in nature, it is suggested to also utilize a case study approach.

## Data Availability Statement

The raw data supporting the conclusions of this article will be made available by the authors, without undue reservation.

## Ethics Statement

The studies involving human participants were reviewed and approved by CLSU-Department of Psychology Ethics Review Committee. Written informed consent to participate in this study was provided by the participants’ legal guardian/next of kin.

## Author Contributions

All authors listed have made a substantial, direct and intellectual contribution to the work, and approved it for publication.

## Conflict of Interest

The authors declare that the research was conducted in the absence of any commercial or financial relationships that could be construed as a potential conflict of interest.

## Appendix A

### Deviant Behavior Variety Scale – Filipino Version

Instruction: Answer the following questions with yes or no. Answer as honestly as possible.

*Panuto: Sagutin ang mga sumusunod na katanungan ng oo o hindi. Sagutin ng may katapatan.*

**Table:**

**During the last year, have you ever… [Sa nakalipas na taon, ikaw ba ay…**]		
	**Yes**	**No**
(1) Been to school or to class after drinking alcohol?^(mi)^ [*Pumasok sa eskwelahan o klase pagkatapos na makainom ng alak?*]		
(2) Lied to adults (e.g., family members, teachers, etc.)?^(mi)^ [*Nagsinungaling sa mga nakakatanda (hal., miyembro ng pamilya, mga guro, at iba pa)?*]		
(3) Used cocaine or heroin?^(si)^ [*Gumamit ng cocaine o heroin?]*		
(4) Used a motorbike or a car to go for a ride without the owner’s permission?^(si)^ [*Gumamit ng sasakyan (motorsiklo o kotse) na walang pahintulot ng may-ari?*]		
(5) Hitted an adult (e.g., teacher, family, security guard, etc.)?^(si)^ [*Nakapanakit ng nakatatanda (hal. guro, pamilya, security guard, at iba pa)?*]		
(6) Used public transport without paying?^(mi)^ [*Sumakay ng pampublikong sasakyan ng hindi nagbabayad?*]		
(7) Damaged or destroyed public or private property (e.g., parking meters, traffic signs, product distribution machines, cars, etc.)?^(si)^ [*Nanira ng pampubliko o pribadong pagmamay-ari (hal.: metro ng tubig, traffic sign, product distribution machines, sasakyan, at iba pa.)?*]		
(8) Used hashish (“hash”) or marijuana (“grass”)?^(mi)^ [*Gumamit ng shabu o marijuana?*]		
9. Stolen something worth more than 1,000 pesos (e.g., in shops, at school, to someone, etc.)?^(si)^ [*Nagnakaw ng isang bagay na nagkakahalaga ng mahigit sa 1,000 pesos (hal. sa shop, sa eskwelahan, sa ibang tao)?*]		
(10) Skipped school for several days without your parents’ knowing?^(mi)^ [*Hindi pumasok ng ilang mga araw nang hindi nalalaman ng iyong magulang?*]		
11. Sold drugs (e.g., hashish, marijuana, cocaine, ecstasy, amphetamines, etc.)?^(si)^ [*Nagbenta ng droga (hal., shabu, marijuana, cocaine, ecstasy, amphetamines, at iba pa)?*]		
(12) Stolen something worth between 100 and 1,000 pesos (e.g., in shops, at school, to someone, etc.)?^(si)^ [*Nagnakaw ng isang bagay na nagkakahalaga ng 100 hanggang 1,000 peso (hal., sa shop, sa eskwelahan, sa ibang tao)?*]		
(13) Skipped classes because you didn’t feel like going, to stay with colleagues, or to go for a ride?^(mi)^ [*Hindi na pumasok dahil hindi mo gusto, para makasama ang mga kaibigan, o para gumala?*]		
(14) Drove a motorbike or a car without having a driver’s license?^(si)^ [*Nagmaneho ng motorsiklo o nang sasakyan ng walang lisensya?*]		
(15) Used LSD “acids,” ecstasy “tablets,” or amphetamines “crystals”?^(si)^ [*Gumamit ng LSD “acids,” ecstasy “tablets,” o amphetamines “crystals”?*]		
(16) Carried a weapon (e.g., knife, pistol, etc.)?^(si)^ [*Nagdala ng sandatang nakamamatay (hal., patalim, baril, at iba pa)?*]		
(17) Stolen something worth less than 100 pesos (e.g., in shops, at school, to someone, etc.)?^(mi)^ [*Nagnakaw ng isang bagay na nagkakahalaga ng mas mababa sa 100 pesos (hal., sa shop, sa eskwelahan, sa ibang tao)?*]		
(18) Done graffiti on buildings or other locations (e.g., school, public transports, walls, etc.)?^(mi)^ [*Nagsulat ng mga graffiti sa mga gusali o iba pang lugar (hal., sa paaralan, mga pampublikong sasakyan, mga pader, at iba pa)?*]		
(19) Broken into a car, a house, shop, school or other buildings?^(si)^ [*Sapilitang pumasok sa sasakyan, sa isang bahay, sa shop, sa eskwelahan at sa iba pang gusali?*]		

*[note: ^(mi)^ items classified as “minor infractions”; ^(si)^ items classified as “serious infractions”]*

### Appendix B

#### Satisfaction With Family Life Scale

Below are five statements with which you may agree or disagree. Using the 1–7 scale below, indicate your agreement with each item by circling the appropriate number on the line following that item. Please be open and honest in responding.

### Appendix C

#### The Big Five Inventory (BFI)

Here are a number of characteristics that may or may not apply to you. For example, do you agree that you are someone who likes to spend time with others? Please write a number next to each statement to indicate the extent to which you agree or disagree with that statement.
